# Multi-frequency sound production and mixing in graphene

**DOI:** 10.1038/s41598-017-01467-z

**Published:** 2017-05-02

**Authors:** M. S. Heath, D. W. Horsell

**Affiliations:** School of Physics and Astronomy, University of Exeter, Stocker Road, Exeter, EX4 4QL UK

## Abstract

The ability to generate, amplify, mix and modulate sound in one simple electronic device would open up a new world in acoustics. Here we show how to build such a device. It generates sound thermoacoustically by Joule heating in graphene. A rich sonic palette is created by controlling the composition and flow of the electric current through the graphene. This includes frequency mixing (heterodyning), which results exclusively from the Joule mechanism. It also includes shaping of the sound spectrum by a dc current and modulating its amplitude with a transistor gate. We show that particular sounds are indicators of nonlinearity and can be used to quantify nonlinear contributions to the conduction. From our work, we expect to see novel uses of acoustics in metrology, sensing and signal processing. Together with the optical qualities of graphene, its acoustic capabilities should inspire the development of the first combined audio-visual nanotechnologies.

## Introduction

Thermoacoustic sound is produced by a material without physical movement. In terms of applications, this has been its principle distinction from other generation mechanisms. It has been observed in many thin film conductors^1–5^ including graphene^6–10^, reduced graphene oxide^11, 12^ and graphene foams^13, 14^. The exceptionally high thermal conductivity^15^ and low heat capacity^16^ of graphene make it the quintessential material for investigating thermoacoustics. Up to now, the thermoacoustic process has been considered too uncontrollable and inefficient to be of scientific or practical interest. The focus has been to improve the efficiency of the transduction. Joule heat in graphene is lost via several routes^17, 18^. How it is lost is not only of fundamental importance but also key to thermal management applications^19, 20^. Loss in the form of light emission has been observed^21–23^. However, the most significant loss is to the substrate supporting the graphene. This is why the drive to greater efficiency has led to attempts to optimise the substrate, the ultimate being to remove it altogether. Practical applications will generally require a substrate, limiting the material choices and resulting efficiency. What has been largely overlooked so far is the role of the electronic properties. This role is the focus of our work.

The Joule heating mechanism acts as an ideal mixer for heterodyne generation. As we will show, heterodynes at the sum and difference of the source frequencies are generated most efficiently when the conductance is linear. (We will see later what happens in the case of nonlinearity). This is in stark contrast to traditional heterodyning systems that require a nonlinear element to mix the input signals (for example, diodes in electronics and nonlinear crystals in optics). Heterodyning is used extensively in telecommunications and signal processing, and has been suggested to be involved in bat echolocation^24^. It is finding new uses in acoustic metrology^25^, gas sensing^26^ and chemical spectroscopy^27^. As such, controllable heterodyning via a linear element will make a mark in a wide variety of fields.

The sound in air resulting from Joule heating can be modelled relatively simply^2^. The sound pressure

1 $$\delta p= {\mathcal E} \frac{fP}{r}\,,$$

where *P* is the power. The linear dependence of this sound pressure on frequency, *f*, is a hallmark of thermoacoustic generation. We assume here that *r*, the distance from the source, is large compared to the lateral size, *L*, of the graphene and that the sound is measured only along the surface normal. Therefore, sound is measured in the far field, *r* > *L* ^2^ *f*/4*v*_a_, and the graphene acts as a point source, *L* ≪ *v*_a_/*f* ^28^. The parameter, *E* contains the thermal properties: ${\mathcal E} =3{e}_{{\rm{r}}}\mathrm{/4}\pi {v}_{{\rm{a}}}^{2}$, where *e*_r_ is the thermal effusivity of the air relative to the whole system and *v*_a_ is the velocity of sound in air. (Full details of the derivation can be found in Supplementary Information Section (SS) 1).

The power dependence of the sound pressure is the key to unlocking the potential of thermoacoustic generation. A current, *I*, driven through a conductor causes a voltage, *V*, to drop across it. The power dissipation, *P* = *IV*, is a result of the Joule heating mechanism. However, the power that appears in equation (1) is not the total power but only the power at frequency *f*. A single ac current source, *I*cos(*ωt*), will result in power *IV*(1 + cos(2*ωt*))/2. This has components at zero frequency (dc) and the second harmonic (2*ω*) of the source. The dc component only heats the device. The 2*ω* component is the source of power for the sound. This is the second hallmark of thermoacoustic generation: an input signal at *f* will cause sound generation only at 2*f*.

**Figure 1:**
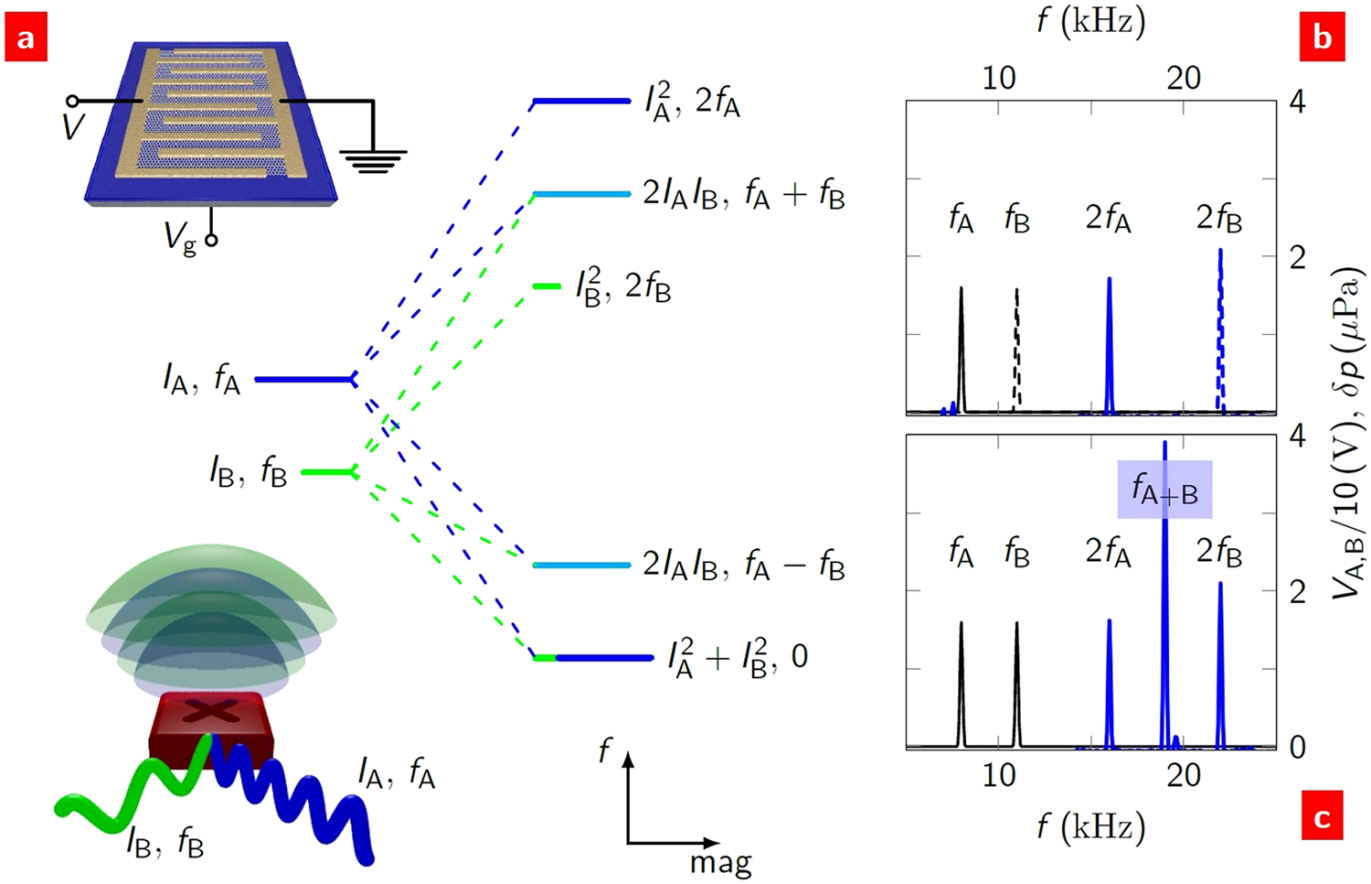
Thermoacoustic generation. (**a**) The sound spectrum (right) resulting from two current sources (left) of differing frequency and amplitude. This is shown schematically in the bottom inset. The frequency and relative magnitude of the sound components are indicated. (The power at each frequency is found by multiplying the relative magnitudes by *R*/2). The heterodynes (cyan) only exist in the presence of both sources. The transistor is shown in the top inset, with electrodes (gold), 6 mm square monolayer graphene (grey), substrate (blue), and electrical connections (black) to the electrodes and gate. (**b**) Acoustic response (blue) to an ac bias voltage *V* (black) in a back-gated graphene field-effect transistor. An individual source at frequency *f*_A,B_ causes a response at the second harmonic 2*f*_A,B_. (**c**) Two frequency sources applied together produce not only the second harmonic responses but also the sum heterodyne frequency at *f*_A+B_ = 19 kHz.

What if the source was driven by a current of more than one frequency? Consider two currents, A and B, with amplitudes *I*_A,B_ and frequencies *f*_A,B_, respectively, as shown in Fig. 1(bottom inset). The power has several components:

2 $$\begin{array}{rcl}P & = & \tfrac{1}{2}R\{({I}_{{\rm{A}}}^{2}+{I}_{{\rm{B}}}^{2})+{I}_{{\rm{A}}}^{2}\,\cos \,\mathrm{(2}{\omega }_{{\rm{A}}}t)+{I}_{{\rm{B}}}^{2}\,\cos \,\mathrm{(2}{\omega }_{{\rm{B}}}t)\\  &  & +2{I}_{{\rm{A}}}{I}_{{\rm{B}}}(\cos ({\omega }_{{\rm{A}}-{\rm{B}}}t)+\,\cos ({\omega }_{{\rm{A}}+{\rm{B}}}t))\}\\  & \equiv  & {P}_{0}+{P}_{{\rm{2A}}}+{P}_{{\rm{2B}}}+{P}_{{\rm{A}}-{\rm{B}}}+{P}_{{\rm{A}}+{\rm{B}}}\,,\end{array}$$

where *ω*_A±B_ ≡ *ω*_A_ ± *ω*_B_ and *R* is the resistance. This result is shown schematically in Fig. 1a. The powers *P*_*i*_ generate sound *δp*_*i*_ at frequency *f*_*i*_. The first three terms are what we expect from a linear superposition of two independent sources. The last two terms are heterodynes: the sum and difference frequencies of the sources.

## Results and Discussion

We present results and analysis of the generation of sound in air from monolayer graphene field-effect transistors (FETs). A schematic of one of our devices is shown in Fig. 1(top inset). To explore a wide range of electrical parameters, we measured a total of 16 back-gated and top-gated FETs, the resistances of which varied in the overall range 10 Ω to 20 k Ω. For all devices, the graphene was etched to a square shape of side *L* = 6 mm. The back-gated FETs were graphene on SiO_2_ (300 nm)/p^+^Si substrates. The p^+^Si formed the back gate electrode, separated from the graphene by the SiO_2_ layer. The top-gated FETs were graphene on quartz substrates. The top gate was formed by a lithium perchlorate-based electrolyte^29–31^. (For full details, see Methods and SS2, 3). Sound pressure above the graphene was measured with a calibrated condenser microphone. We used a lock-in technique to resolve both the magnitude and phase (relative to the source) of the sound (SS4 and Figure S1). This technique gave a sufficiently high signal-to-noise ratio that acoustic isolation of the system was not required.

To facilitate ease of comparison later, it will often be useful to normalise the sound pressure by one or more of the parameters in equation (1). To indicate this, the parameter(s) of normalisation will appear as a superscript to *δp*. For instance, normalisation by the distance *δp* ^*r*^ ≡ *δpr*, frequency, *δp* ^*f*^ ≡ *δp*/*f* and power *δp* ^*P*^ ≡ *δp*/*P* allow ease of comparison of data taken at different distances, frequencies and powers without further analysis. Bias- and resistance-normalisation will also be used when full normalisation by the power is not appropriate.

### Second harmonic generation

**Figure 2:**
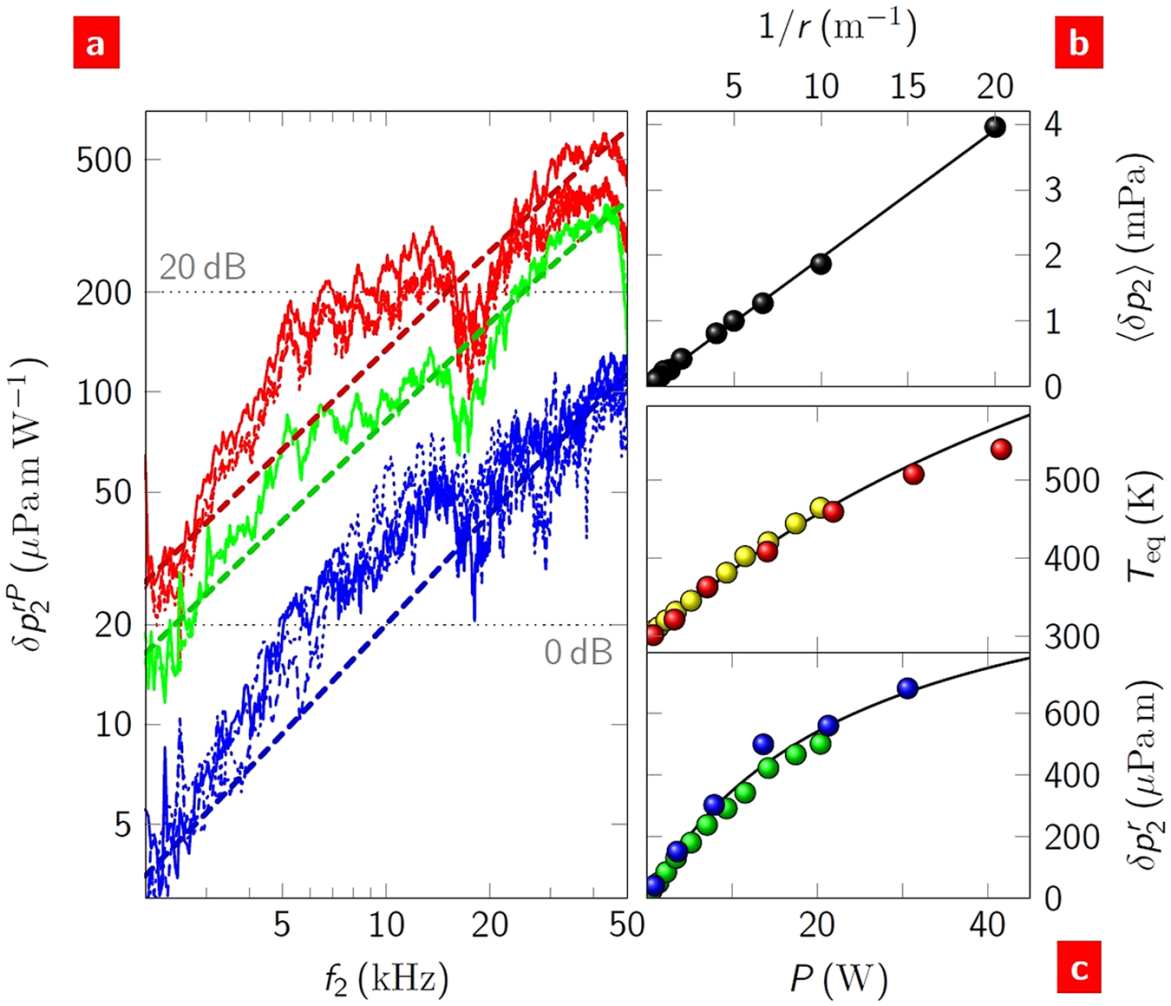
Second harmonic generation. (**a**) Sound pressure spectra from a range of devices (measured at *r* = 50 mm). The bottom blue group are spectra from four back-gated FETs. The top red group are spectra from four top-gated devices before the electrolyte was deposited. The middle green group are spectra from two top-gated FETs after electrolyte deposition. Different line types designate device data within groups. In all cases, *V*_g_ = 0. The expected dependences from equation (1) are shown as dashed lines of the same colour as the data. Indicative sound pressure levels, 20log(*δp* ^*rf*^/*δp*_ref_), where *δp*_ref_ = 20 *μ* Pa m kHz^−1^, are shown as horizontal dotted lines. (**b**) Sound pressure from a back-gated FET with a source power of 3.4 W as a function of inverse device-microphone separation. The sound pressure has been averaged over the frequency range 20–50 kHz. (**c**) (Top) Device temperature measured in two back-gated FETs (differentiated by colour) and calculated (line) as a function of source power. (Bottom) Sound pressure at *f*_2_ = 40 kHz (measured at *r* = 50 mm) for the same devices. The solid line is the expected dependence from equation (1).

Second harmonic sound generation results from a source driven by a single frequency ac current. An example of this is shown in Fig. 1b. We used this to investigate the individual components of equation (1). Figure 2a shows the sound pressure spectra resulting from an ac bias voltage for both back- and top-gated FETs. The predicted sound pressure spectra from equation (1) are shown as dashed lines. The parameters used in the equation were either experimentally determined or established material properties (SS5 and Table S1). The thermal properties contained within *E* dictate the absolute magnitude of the sound. The back-gated FETs have relatively large substrate effusivity, ∼10^4 ^JK^−1^m^−2^s^−0.5^ (compared to values of ∼10 and 10^3^ for air and quartz, respectively; Figure S2), and produced the quietest response; the loudest response was produced by graphene on quartz substrates (the top-gated FETs before the electrolyte was deposited). The overall linear dependence of *δp*_2_ on frequency verified the thermoacoustic nature of the signal and allowed us to calculate *δp* ^*f*^. The fine structure in the sound spectra was fully reproducible and found to be the result of phase variation as the sound wavefronts pass the microphone surface (SS6 and Figure S3). Figure 2b shows *δp*_2_ as a function of inverse-separation. The linear increase with 1/*r* is that expected for a point source. By verifying this, it was possible to calculate *δp* ^*r*^ from measurements made at any separation within the experimental range.

The power dependence of the sound is more subtle than the frequency and separation dependences. Figure 2c shows this dependence for the equilibrium device temperature, *T*_eq_, and the sound pressure. The back-gated FETs were found to reach high temperatures at high ac biases. This allowed us to explore the mechanisms of heat loss other than sound from the devices. The temperature was found using a calibrated thermal camera, Fig. 2c(top). To a good approximation for powers up to 20 W, *T*_eq_ = *aP*+*T*_0_, where *a* ≈ 6 K/W and *T*_0_ = 293 K. This temperature is a result of the Joule heat produced in the device balanced by the convective and radiative heat losses to the surrounding air. The expected dependence (SS7) is shown in the figure (and Figure S4). A fit of the theory to the sound pressure is shown in Fig. 2c(bottom). The sublinearity at high powers is accounted for through the effect of the equilibrium temperature on the speed of sound in air immediately above the graphene (SS5). For the following experiments, we kept the power low (<10 W) to be in the linear regime and to calculate *δp* ^*P*^.

### First harmonic generation

By adding a dc current to the ac current, sound can be generated at the same frequency as the source. This first harmonic generation can be seen in Fig. 1a to result from the combination of the sum and difference heterodynes (where *f*_B_ = 0). The second harmonic from the ac current remains; the dc current feeds into the dc power loss, *P*_0_. Therefore, the device generates both second and first harmonic sound simultaneously. If the magnitudes of the ac and dc currents are equal, the first harmonic sound, *δp*_1_, is four times larger than the second harmonic. This is not of particular note in itself, but its linear dependence on the dc current is. To explore this, we recast the expression for the power as *P*_1_ = 2*V*_dc_ *V*_ac_/*R* to clearly define the dc and ac components.

**Figure 3:**
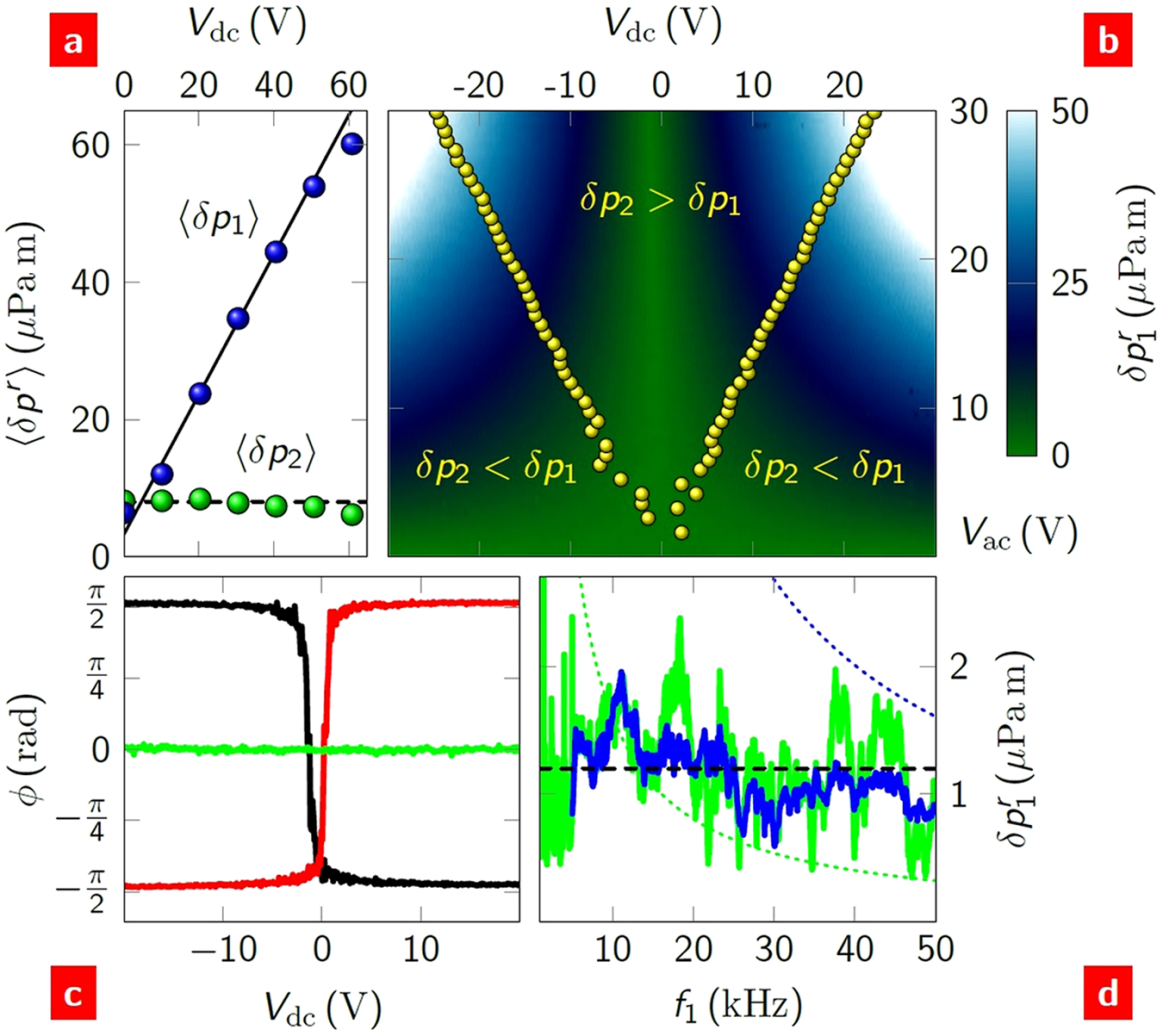
Bias control of the first harmonic generation. (**a**) Sound pressure measured at the first (blue) and second (green) harmonics of the source frequency as a function of dc bias across a back-gated FET. The sound pressure values are averaged over the frequency ranges *f*_*i*_ = 38–42 kHz, *i* = 1, 2. The solid line is a linear fit to the first harmonic data; the dashed line is 〈*δp*〉 = 8*μ*Pa. *P*_2_ was fixed at 0.1 W for all measurements. (**b**) The first harmonic sound as a function of both ac and dc bias at *f* = 12 kHz. The second harmonic sound increases quadratically with *V*_ac_: the yellow symbols indicate where, experimentally, *δp*_1_ = *δp*_2_. (**c**) The phase of the first (black at *f*_1_ = 12 kHz; red at *f*_1_ = 15 kHz) and second (green) harmonic as a function of dc bias. (**d**) Example of a flat first harmonic sound spectrum (solid lines) created by decreasing the dc bias as *V*_dc_ = *V*_ref_(*f*_ref_/*f*), where *V*_ref_ = 10 V at *f*_ref_ = 1 kHz. The ac bias was fixed at 10 V. The applied dc biases are shown (in Volts) as dotted lines in colours corresponding to the sound spectra, which have been normalised to *V*_ref_ for comparison.

The expression for the power at the first harmonic indicates that the sound at this harmonic can be amplified by increasing the dc bias. Figure 3a shows the sound as a function of dc bias at a fixed ac bias of 20 V. We see a linear increase of *δp*_1_ and a flat response of *δp*_2_. For sound reproduction, this is ideal as the second harmonic can be kept below the threshold of hearing (20 *μ*Pa) and ‘volume’ of the first harmonic can be tuned by the dc bias.

The balance between the two harmonic sounds can be achieved by varying the experimental parameters. Figure 3b shows *δp*_1_(*V*_dc_, *V*_ac_). The magnitude of the first harmonic increases linearly with both *V*_ac_ and |*V*_dc_| and is roughly symmetric about *V*_dc_ = 0. In contrast, *δp*_2_ has no dependence on *V*_dc_, Fig. 3a, but increases quadratically with *V*_ac_. (In fact, as will be shown later, *δp*_2_ does have a small dependence on *V*_dc_). As a result, for a given {*V*_dc_, *V*_ac_} either the first or second harmonic can dominate. The regions are delineated in the figure. The boundary *δp*_1_ = *δp*_2_ is linear, as expected from equations (1) and (2).

The boundary between first and second harmonic sound dominance is frequency dependent. We investigated this dependence explicitly for the phase of both harmonics. Figure 3c shows the phase of the first and second harmonics as a function of dc bias. The second harmonic behaves as we expect: like the magnitude, the phase is independent of dc bias. In contrast, irrespective of frequency, the phase of the first harmonic switches by half a cycle around *V*_dc_ = 0. This can be seen to result from the linear dependence of *δp*_1_ on this bias. The direction of the switch does depend on frequency: by changing the frequency through one period, Δ(1/*f*) = *r*/*v*_a_, (SS6) its direction about *V*_dc_ = 0 is reversed.

The dc bias can be used to arbitrarily shape the sound spectrum. For example, to create a white (flat) sound spectrum, the dc bias must be inversely proportional to the frequency. Experimentally, by applying such a bias we observe this white spectrum from 1 to 50 kHz, Fig. 3d. By minimising the fine structure (an experimental artefact), this example alone could easily find use as a calibration sound source.

### Heterodyne generation

**Figure 4:**
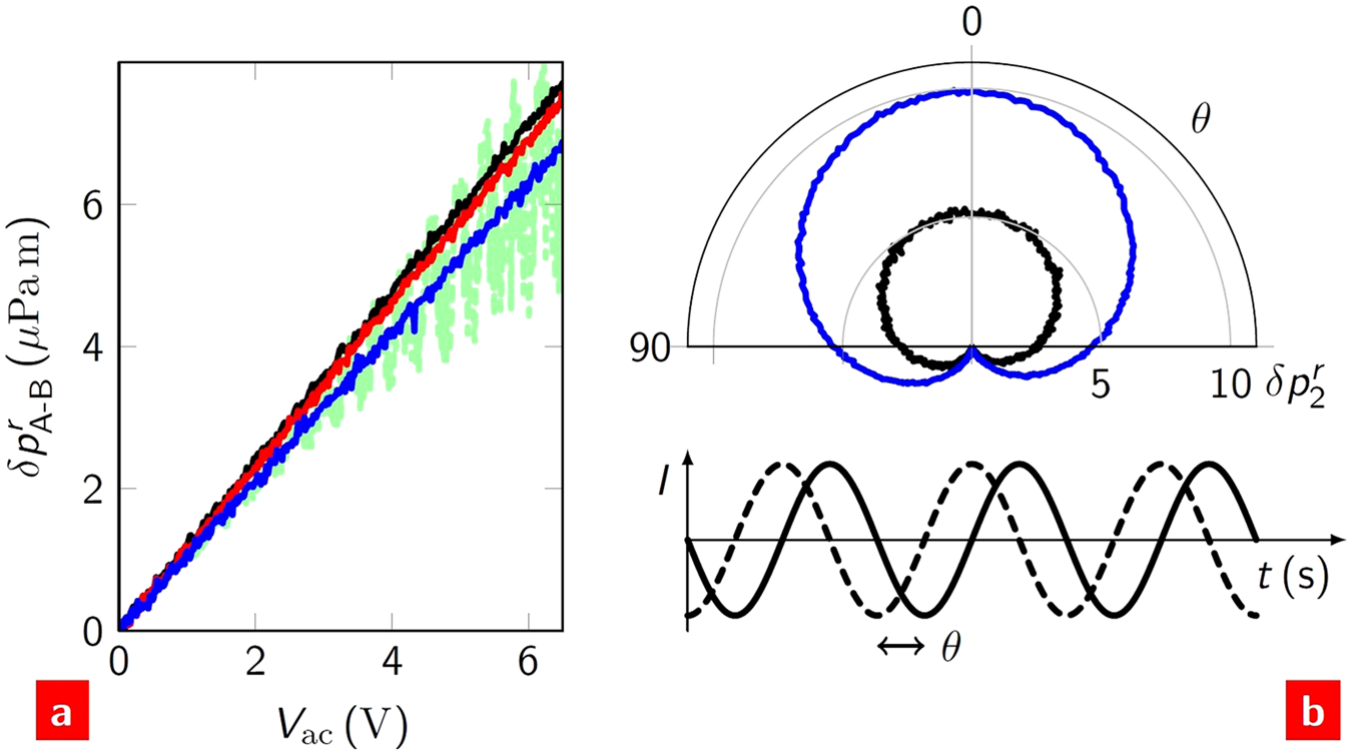
Heterodyning. (**a**) The acoustic difference heterodyne, |*f*_A_−*f*_B_| = 16 kHz, as a function of the ac bias magnitude of source A. Source B had a fixed magnitude equal to the maximum of A. Different colours correspond to different source A frequencies: *f*_A_ = 1 kHz (black), 10 kHz (red) and 100 kHz (blue). The green, dashed curve shows *f*_A_ = 8 kHz. (**b**) Homodyne sound generation as a function of the phase shift, *θ*, between A and B: *f*_A,B_ = 5 kHz (black) and 10 kHz (blue). The phase shift between A and B is shown schematically below.

By sourcing ac currents at two frequencies, acoustic heterodynes can be generated at the sum and difference of these frequencies. The power at the heterodynes,

3 $${P}_{{\rm{A}}\pm {\rm{B}}}={I}_{{\rm{A}}}{I}_{{\rm{B}}}R,$$

is a simple combination of the current amplitudes and resistance. We can use equation (3) to test the ideal mixing expected from the Joule heating mechanism. An example of the acoustic sum heterodyne is shown in Fig. 1c. Figure 4a shows that if the frequency difference between the sources is maintained then the sound pressure at the heterodyne is independent of the absolute values of the source frequencies and increases linearly with the bias. (The slight suppression with increasing *f*_A_ is due to capacitive loss). The only observed deviation from this behaviour occurs when the heterodyne coincides with the second harmonic frequency of one of the sources. In this instance, a quadratically increasing envelope of the pressure occurs with increasing bias (see Methods).

Homodyning occurs when the two source frequencies are equal. Homodynes are sensitive to the phase shift between the sources. As such, they are commonly used in optical and acoustic detection systems. In thermoacoustics, the two sources generate sound only at the second harmonic. This can be seen from Fig. 1a. The sum heterodyne combines with the second harmonics of the individual sources; the difference heterodyne adds to the dc power loss. Figure 4b shows the second harmonic sound as a function of the phase difference between the sources. Although the contribution of this phase in equation (2) appears rather complex, the effect on the magnitude of the sound is simple: if the phase difference is zero, the sound is maximised; if it is half a cycle, the sound is turned off. The sensitivity of the homodyne sound to this phase would make it useful as a detector of electronic phase changes in one of the sources.

### Sound gating

**Figure 5:**
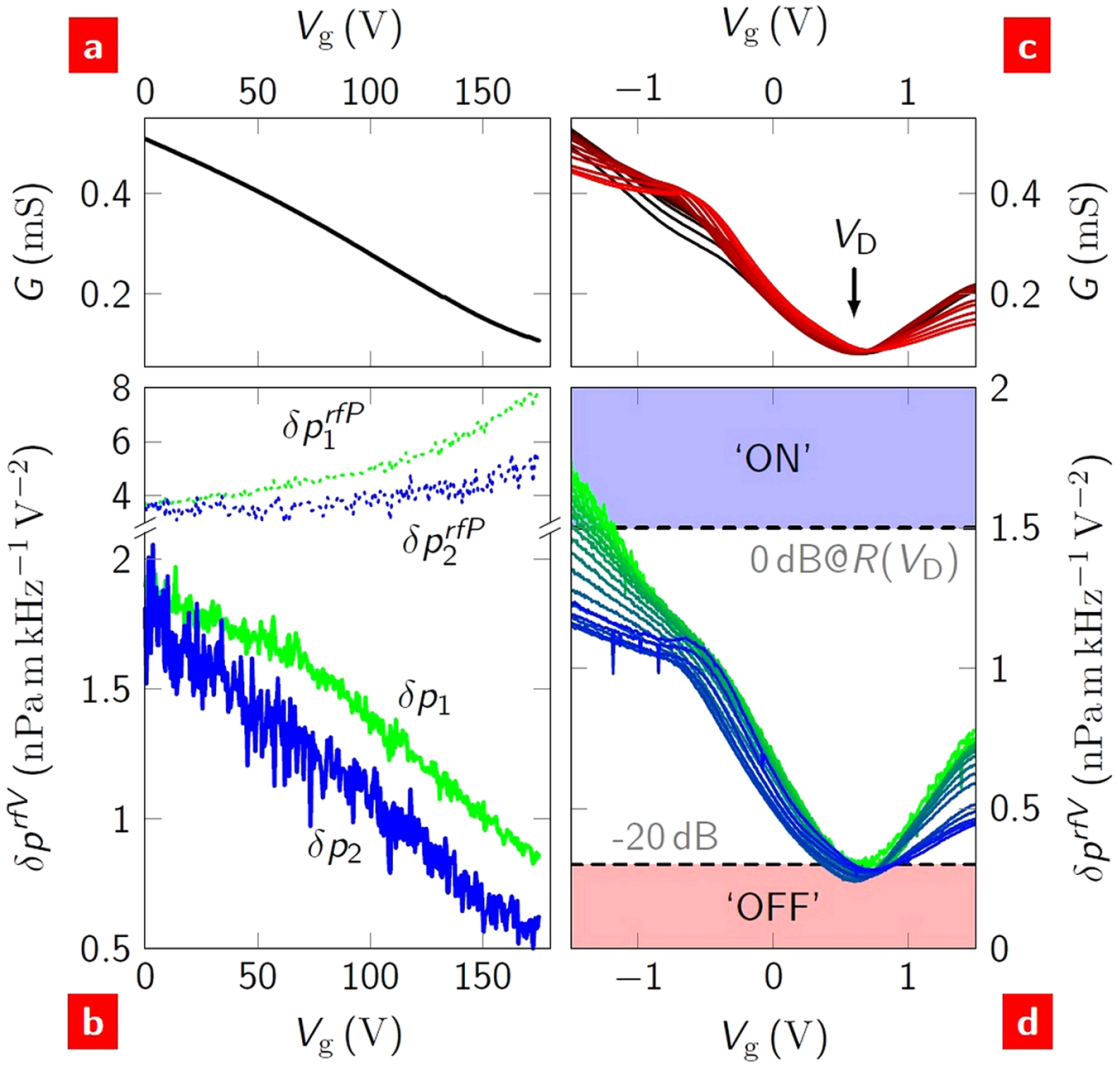
Sound gating with a field-effect transistor. (**a,b**) A back-gated FET. The conductance (**a**), and the first (solid green) and second (solid blue) harmonic sound (**b**) as a function of the back-gate voltage. The power-normalised sound pressures are shown as dotted lines: the units for *δp* ^*rfP*^ are *μ* Pa m kHz^−1^W^−1^. (**c,d**) A top-gated FET. Conductance (**c**) and second harmonic sound (**d**) as a function of top-gate voltage measured at *f*_1_ = 18 kHz for different source powers from 0.05 W (black/green) to 0.5 W (red/blue). The ‘on’/‘off’ state of the sound, described in the main text, is indicated by the blue/red shaded regions, respectively.

Beyond its thermal properties, graphene plays two further roles in the sound generation. First, its electrical properties can be tuned by the transistor gate. This tuning could be used to switch or modulate the sound output. Second, it allows us to invert our original question: could we use the sound generation to reveal something about the conduction in graphene? Gate control of the sound output is possible in a field-effect transistor. The resistance and sound were measured by applying a gate voltage, *V*_g_, between the graphene and the gate electrode. Figure 5a,b shows measurements of the conductance, *G* = 1/*R*, and sound pressure as a function of *V*_g_ for a back-gated FET (see Methods). As *V*_g_ increases, the conductance decreases, approaching a minimum at *V*_g_ = *V*_D_. In the limit *P* → 0, this minimum occurs at ∼140 V, which is coincident with the Dirac point (the energy at which the conduction and valence bands meet). In order to observe and compare the explicit dependence of *δp*_1,2_ on *R*(*V*_g_), they were normalised by the applied biases (see equation (2)): $\delta {p}_{1}^{V}\equiv \delta {p}_{1}\mathrm{/(2}{V}_{{\rm{dc}}}{V}_{{\rm{ac}}})$; $\delta {p}_{2}^{V}\equiv \delta {p}_{2}/({V}_{{\rm{ac}}}^{2}\mathrm{/2)}$. It can be seen that both harmonics are indeed proportional to 1/*R*, as predicted. As a result, the magnitudes and relative phases of the sounds generated at *f*_1_ and *f*_2_ can be completely specified by the set {*V*_dc_, *V*_ac_, *V*_g_}.

The gate control could be used to switch the sound on and off. Figure 5c shows *G*(*V*_g_) for a top-gated device for powers from 0.05 W to 0.5 W (see Methods). The conductance minimum is seen to occur at *V*_D_∼0.6 V and this shifts with increasing power by ∼0.05 V over the power range considered. The two branches about *V*_D_ do not vary up to 0.2 W; at higher powers they typically become less conductive. As with the back-gated FETs, the sound pressure varies in a similar way to *G*(*V*_g_), so for a fixed bias the gate can be used to effectively switch the sound on and off by toggling its voltage between *V*_g_ = *V*_D_ and *V*_g_ = *V*_D_ − 2 V, Fig. 5d. The ‘on’ and ‘off’ state we define at *P* = 1 W as being at sound pressure levels, 20log(*δp* ^*rf*^/*δp*_ref_), of 0 dB and −20 dB, respectively. These correspond to ‘at’ and (an order of magnitude) ‘below’ the limit of human hearing at *f* = 1 kHz and *r* = 1 m, where *δp*_ref_ = 20 *μ* Pa m kHz^−1^.

### Nonlinear conduction in graphene

The sound generation and gate control can be used to investigate the conduction mechanisms in the graphene. If the bias-normalised sound pressure in Fig. 5b is further normalised by the resistance, it should be constant as a function of gate voltage. However, *δp* is found to be enhanced as the gate voltage approaches *V*_D_. This is possible, if the charge transport in graphene has a nonlinear component. To second order in the current, the voltage

4 $$V(t)={R}_{0}{I}_{{\rm{dc}}}+{R}_{1}{I}_{{\rm{ac}}}\,\cos (\omega t)+\tfrac{1}{2}{R}_{2}{I}_{{\rm{ac}}}^{2}{\cos }^{2}(\omega t)\,,$$

where *R*_0_ = *V*_dc_/*I*_dc_ is the dc resistance, *R*_1_ = d*V*/d*I* is the differential resistance, and *R*_2_ = d^2^ *V*/d*I* ^2^ is the second-differential resistance. Up to this point, we have assumed *R*_0_ = R_1_ ≡ *R*: experimentally, as *R*_2_ *I*_ac_ < *R*_1_ we continue with this assumption. As a result, the source power has additional terms of 3*R*_2_(*I*_ac_/2)^3^ and *R*_2_ *I*_dc_(*I*_ac_/2)^2^ for *P*_1_ and *P*_2_, respectively (SS8). These power components can account for the differences seen in the dependences of *δp*_1_ and *δp*_2_ on *V*_g_, if |*R*_2_| increases with *V*_g_.

**Figure 6:**
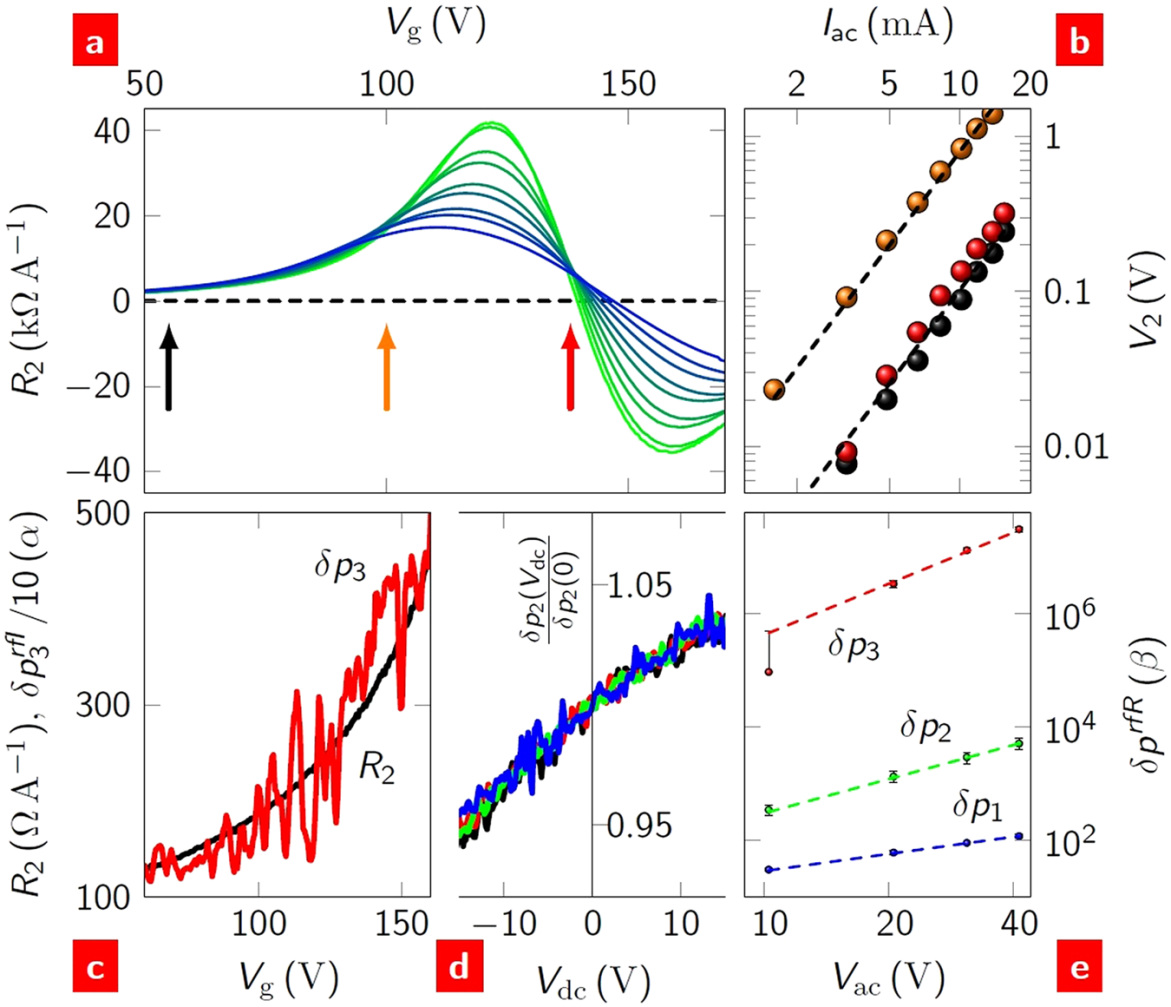
Third harmonic generation. (**a**) Second-differential resistance as a function of gate voltage of a back-gated FET immersed in liquid helium (*T* = 4.2 K). The curve colour ranges from green at *P* = 0.007 W to blue at *P* = 0.7 W (not in equal steps). (**b**) The voltage drop across the device at *f*_2_ as a function of ac bias. The data (symbols) are taken at the gate voltages indicated by identically coloured arrows in **a**; the dashed lines are the expected ${I}_{{\rm{ac}}}^{2}$ dependence. (**c**) *R*_2_(*V*_g_) in ambient conditions at *P* = 0.7 W shown together with the simultaneously measured current-normalised third harmonic sound pressure, $\delta {p}_{3}^{I}=\delta {p}_{3}/{({I}_{{\rm{ac}}}\mathrm{/2)}}^{3}$ (measured at *f*_3_ = 42 kHz and *r* = 25 mm). $\alpha \equiv \mu {\rm{Pa}}\,{\rm{m}}\,{{\rm{kHz}}}^{-{\rm{1}}}\,{{\rm{A}}}^{-{\rm{3}}}$. (**d**) *δp*_2_ as a function of dc bias, normalised to its value at *V*_dc_ = 0. Curves at four ac biases: 10 V (black), 20 V (red), 30 V (green) and 40 V (blue). (**e**) First, second and third harmonic sound pressures as a function of ac bias. Each datum point is an average over a range of frequencies 10 < *f* < 14 kHz and dc biases −34 < *V*_dc_ < +34 V. The dashed lines are the predicted ${I}_{{\rm{ac}}}^{n}$ dependences: *n* = 1 (blue), *n* = 2 (green) and *n* = 3 (red). $\beta \equiv \mu {\rm{Pa}}\,{\rm{m}}\,{{\rm{kHz}}}^{-{\rm{1}}}\,{{\rm{\Omega }}}^{-{\rm{1}}}\,{{\rm{A}}}^{{\rm{2}}-{\rm{n}}}$.

If the second-differential resistance is the origin of the enhancement then sound will be generated at the third harmonic. The power at this harmonic,

5 $${P}_{3}={R}_{2}{({I}_{{\rm{ac}}}\mathrm{/2})}^{3},$$

depends exclusively on *R*_2_. An explicit measurement of *R*_2_ was made along with this predicted harmonic component. Figure 6a shows *R*_2_(*V*_g_) of a back-gated FET for different source powers. In this experiment only, the device was immersed in liquid helium to distinguish the effect of bias from the effect of an increase in *T*_eq_ (Figure S4). That it is *R*_2_ and not an artefact of the resistance is shown in Fig. 6b, where the voltage drop across the channel of the FET at the second harmonic of the source frequency (*V*_2_) is shown to have the quadratic dependence on the source current (equation (4)). The origin of *R*_2_(*V*_g_) is not important for the present discussion. (Various types of nonlinear behaviour have been predicted^32–34^ and observed^17, 35–37^). The significance here is that *R*_2_ is finite and increases in magnitude as the gate voltage approaches the Dirac point. Figure 6c–e shows that this does indeed account for the observed enhancement of the sound pressure: Fig. 6c shows the direct correlation between *R*_2_ and *δp*_3_; Fig. 6d shows the dependence of *δp*_2_ on *V*_dc_; and Fig. 6e shows the dependence of each harmonic on the ac bias. For Fig. 6e, the wide range of dc biases over which the data are averaged, and the fact that ${R}_{{\rm{2}}}{I}_{\mathrm{dc},\mathrm{ac}}\ll {R}_{1}$ means that the predicted power-law dependence on ${V}_{{\rm{ac}}}^{n}$ is *n* = 1, 2, 3 for *δp*_1,2,3_, respectively. The *δp*_2_(*V*_dc_) dependence shown in Fig. 6d can be used to estimate a value for *R*_2_ of ∼+100 ΩA^−1^ at *V*_g_ = 0, which is comparable to that measured directly in the charge transport. For sufficiently large values of *R*_2_ beyond our current experimental range, the second harmonic acoustic response could be turned off altogether.

In summary, we have demonstrated a highly versatile thermoacoustic sound generator ranging from audible to ultrasonic frequencies. The most significant result of our work was to show that the Joule heating mechanism in graphene controllably mixes frequency components of a current source together. This not only has applications in acoustics but also in signal processing where it could be used to create an acoustically-coupled, linear electronic mixer. We further showed the simplicity of this mixing in heterodyning, homodyning, amplification and equalisation. In addition to modulation achieved using a transistor gate, this afforded full control over the sound output. Such a generator has a wide range of potential applications, from multiplexing in telecommunications to calibrated sound sources for metrology and sensing. One of our most intriguing results was that this generation can be used to quantitatively measure the conduction properties of graphene. Nonlinearity in the conduction has important consequences for optical, electronic and thermal applications of this material so our acoustic probe will provide fresh insights in these areas.

## Methods

### Device fabrication

Back-gated graphene FETs were created from CVD-grown monolayer graphene transferred to a degenerately doped silicon substrate coated by 300 nm silicon dioxide (SS2). The electrodes were formed of 50 nm thick Au thermally evaporated directly onto the graphene. A thin layer of Cr was used to anchor the extensions of these electrodes on the silicon dioxide surface. The graphene–gold interfacial contact resistance, determined to be ∼0.45 Smm^−2^, was sufficiently low (<3% of the total resistance) that it could be neglected (SS3). Four devices had 12 electrodes spaced around the edge of the graphene; other devices had two interdigitated electrodes separated by gaps of either 200 *μ*m or 100 *μ*m (Figure S1). Silver paint was used to fix the device to the base of a ceramic chip carrier. The paint allowed electrical connection to the Si and, therefore, to measure the device as a field-effect transistor by applying a gate voltage between the silicon and the graphene.

Top-gated FETs were created from CVD-grown monolayer graphene transferred to quartz substrates. A shadow mask technique was used to evaporate four Au/Cr electrodes on the corners of the graphene (a Van der Pauw geometry) and an additional electrode for the gate. All devices were nominally identical. The gate was formed by drop casting a lithium-based electrolyte layer onto the surface of the graphene. The electrolyte source material was created by mixing Poly(Ethyl Oxide) with Lithium Perchlorate (in an 8:1 ratio) in methanol, ultrasonicating the solution for 1 hour, then centrifuging at 10,000 rpm for 5 min. The supernatant was then drop cast by pipette onto the graphene.

### Thermoacoustic measurements

The ac voltage source was that of a lock-in amplifier (Signal Recovery 7265), amplified by a high voltage amplifier. The sound generation was measured using a calibrated condenser microphone (Earthworks M50), which had a constant sensitivity across the full spectral range of the experiment, at distances from 50 mm to 2 m away from the device (SS4 and Figure S1). The output signal from the microphone was amplified (Earthworks ZDT1022) and measured by lock-in amplifiers referenced to the source frequency. For the dc bias measurements, a floating dc voltage source (IOtech DAC488) was connected in series with the ac source. The full spectral response was additionally measured using a spectrum analyser (Stanford Research Systems SR785). For heterodyning, sources were added together (prior to the high voltage amplification) using a differential amplifier (Femto DLPVA-100). To measure the magnitude of the heterodyne signal, the lock-in amplifier was locked to the signal from the microphone at the heterodyne frequency (*i.e*. it uses the measured signal frequency as a reference as there is no real source at this frequency). The apparent periodicity seen in Fig. 4a is a temporal aliasing artefact caused by this different method of measurement.

The sound gating of the back-gated FETs required large voltages to be applied. To avoid breakdown of the gate dielectric in air, which would normally occur above |*V*_g_| ∼ 30 V, a steady stream of helium gas was directed across the device throughout. With this stream, the breakdown voltage was extended to ∼180 V. In top-gated FETs, the electrolyte forming the gate fails at *V*_g_ ∼ 2 V^31^. As the resistances of the top-gated devices ranged from 2–200 kΩ, biases much greater in magnitude than this voltage were required to source sufficient power to generate sound. To apply such biases, we used the fact that the ions in the electrolyte are slow to respond to changes in the applied gate voltage: the maximum slew rate, Δ*V*_g_/Δ*t*, calculated from transient response measurements was found to be ∼10^−2^ V s^−1^. The rate of source signal change (10^4^–10^6^ V s^−1^) is much higher than this slew rate and, as a result, ac source–drain biases well over an order of magnitude larger than the gate voltage range could be applied to the channel without any gating artefacts.

### Electrical and thermal measurements

The differential resistance was determined by measuring the voltage drop across the device together with the voltage drop across a ballast resistor (1 kΩ, 50 W) in series with the device. These were measured simultaneously by lock-in amplifiers referenced from the source. Surface temperatures were measured using a thermal imaging camera (FLIR E6). Calibration of the camera was performed by measuring the surface temperature of a device in a calibrated heating stage (Linkam THM600) at a number of fixed stage temperatures.

## Electronic supplementary material

Multi-frequency sound production and mixing in graphene: supplementary information
