# Supplementary material for: Multi-frequency sound production and mixing in graphene
Source: Sci Rep. 2017 May 2;7:1363. doi: 10.1038/s41598-017-01467-z (PMC5430977; doi:10.1038/s41598-017-01467-z)
Supplement: Supplementary file 1 — Multi-frequency sound production and mixing in graphene: supplementary information [file 41598_2017_1467_MOESM1_ESM.pdf]

Multi-frequency sound production and mixing in graphene: supplementary information

M. S. Heath and D. W. Horsell
School of Physics and Astronomy, University of Exeter, Stocker Road, Exeter, EX4 4QL, U.K.

Model of the thermoacoustic generation—details of the device fabrication—calculation of the contact resistance—measurement circuit—parameters used in the fits—spectral analysis—calculation of the device temperature—nonlinearity and heterodyning.

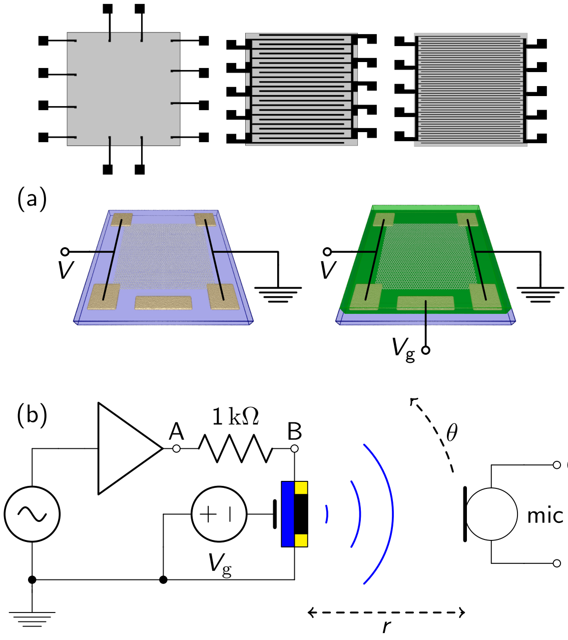

FIG. S1. (a) Top: various electrode geometries used in the back-gated FETs. Electrodes (black) and graphene (grey; $6 \times 6\,\mathrm{mm}^2$) are shown for peripheral (left), $200\,\mu$m (middle) and $100\,\mu$m (right) interdigitated electrodes. Bottom: top-gated FETs on quartz, before (left) and after (right) electrolyte deposition. The electrolyte is shown as a green layer. The gate electrode is shown at the front edge. (b) Schematic of the measurement circuit, showing the microphone (mic) placed at a distance r from the FET channel.

S1. THERMOACOUSTIC RESPONSE

Our model is based largely on that of Ref. 1. We include a short derivation here to show the logic of applying the model to our particular devices.

The acoustic wave generated in the air is considered as a closed system of volume $\mathcal{V}_\mathrm{a}$ that transports energy at velocity v_a, the speed of sound. From the first law of thermodynamics, we have

$$\delta U = \delta Q + \delta W \ ,$$

where U is the mean internal energy, Q is the heat supplied to the system and W is the work done on it. For thermoacoustic devices, $\delta W = 0$. Therefore, the variation in air pressure measured in experiment,

$$\delta p = \frac{\delta U}{\mathcal{V}_\mathrm{a}} = \frac{\delta Q_\mathrm{a}}{\mathcal{V}_\mathrm{a}} \ . \qquad \text{(S1)}$$

It remains to find δQ_a and $\mathcal{V}_\mathrm{a}$. The heat put into the device is that of the Joule heating:

$$\delta Q_\mathrm{in} = \delta Q_\mathrm{g} + \delta Q_\mathrm{a} + \delta Q_\mathrm{s} = P_2/f_2 \ ,$$

where δQ_g is the heat stored in the graphene (g), and $\delta Q_\mathrm{a} + \delta Q_\mathrm{s}$ is the heat out of the graphene into the air (a) and substrate (s). We consider here only a single ac source such that it is the power at the second harmonic, P_2, that is the source of the air pressure fluctuations δp. Now,

$$\delta Q_\mathrm{a} = \frac{C_\mathrm{a}}{C_\mathrm{g} + C_\mathrm{a} + C_\mathrm{s}} \delta Q_\mathrm{in} \ ,$$

where $C_{\mathrm{g(s)}}$ is the heat capacity of graphene (substrate) and C_a is the isobaric heat capacity of air.

If we consider the graphene as a film of area S_g and thickness $d_\mathrm{g} = 0.34\,\mathrm{nm}$ then its heat capacity

$$C_\mathrm{g} = c_\mathrm{g} \rho_\mathrm{g} S_\mathrm{g} d_\mathrm{g} \ ,$$

where ρ_g is its density and c_g its specific heat capacity. Similarly,

$$C_{\mathrm{a(s)}} = c_{\mathrm{a(s)}} \rho_{\mathrm{a(s)}} S_\mathrm{g} d_{\mathrm{a(s)}} \ ,$$

where the same area is considered but

$$d_{\mathrm{a(s)}} = \sqrt{\frac{\kappa_{\mathrm{a(s)}}}{c_{\mathrm{a(s)}} \rho_{\mathrm{a(s)}} \omega_2}}$$

is the skin depth of the thermal boundary layer in the air (substrate) and κ is the thermal conductivity. We can now recast the thermal capacities in terms of material and experimental parameters:

$$C_{\mathrm{a(s)}} = \sqrt{\frac{\kappa_{\mathrm{a(s)}} c_{\mathrm{a(s)}} \rho_{\mathrm{a(s)}}}{\omega_2}} S_\mathrm{g} = e_{\mathrm{a(s)}} S_\mathrm{g} \omega_2^{-1/2} \ ,$$

where e is the thermal effusivity. We can write

$$\delta Q_\mathrm{a} = \frac{e_\mathrm{a}}{e_\mathrm{a} + e_\mathrm{s} + e_\mathrm{g}^*} \delta Q_\mathrm{in} = e_\mathrm{r} \frac{P_2}{f_2} \ ,$$

where we have defined, for convenience, the effusivity of air relative to the effusivity of the whole system,

$$e_{\mathrm{r}} = \frac{e_{\mathrm{a}}}{e_{\mathrm{a}} + e_{\mathrm{s}} + e_{\mathrm{g}}^*}$$

and $e_{\mathrm{g}}^* \equiv c_{\mathrm{g}}\rho_{\mathrm{g}}d_{\mathrm{g}}\sqrt{\omega_2}$ as an effusivity-like term for graphene.

To find $\mathcal{V}_{\mathrm{a}}$ we assume our device is a point source: $L \ll v_{\mathrm{a}}/f_2$. In this case, during the time $1/f_2$ the energy is contained in a hemisphere of volume

$$\mathcal{V}_{\mathrm{a}} = \frac{2}{3}\pi\left(\frac{v_{\mathrm{a}}}{f_2}\right)^3 ,$$

with the pressure maximum occurring at $r_0 = v_{\mathrm{a}}/2f_2$. We can now write Eqn. S1 including attenuation as

$$\delta p(r, f_2) = \delta p(r_0, f_2)\frac{r_0}{r}\mathcal{A} ,$$

where $\mathcal{A} \equiv \exp(-\alpha(r - r_0))$,

$$\alpha = \frac{\omega_2^2\eta}{2\rho_{\mathrm{a}}v_{\mathrm{a}}^3}\left(\frac{4}{3} + \frac{\gamma - 1}{\mathrm{Pr}}\right)$$

is the classical (viscous plus thermal) absorption coefficient under the Stokes assumption, $\eta_{\mathrm{B}} = 0$, γ is the ratio of heat capacities of air, $\mathrm{Pr} = \eta c_{\mathrm{a}}/\kappa_{\mathrm{a}}$ is the Prandtl number and η is the shear viscosity.

The final, full expression for the sound pressure must also include a factor that accounts for the angular position of the microphone with respect to the surface normal of the graphene. The directional factor[2]

$$\mathcal{H} = |\mathrm{sinc}(\pi f_2 L \sin(\theta)/v_{\mathrm{a}})\mathrm{sinc}(\pi f_2 L \sin(\varphi)/v_{\mathrm{a}})| ,$$

where (θ, φ) are orthogonal angles of detection with respect to the surface normal of the device. The resulting sound pressure

$$\delta p = \mathcal{H}\mathcal{E}\mathcal{A}\frac{f_2 P_2}{r} , \qquad \text{(S2)}$$

where $\mathcal{E} \equiv 3e_{\mathrm{r}}/4\pi v_{\mathrm{a}}^2$. We verified the dependence of $\mathcal{H}$ on (θ, φ) and, for all experiments presented, set these angles to zero.

To explicitly check whether our devices were acting as point sources, we measured the sound pressure spectrum from devices with reduced dimensions (L) down to 1 mm. The result was identical to that for the larger devices, both in magnitude and structure, from which we concluded that any finite-size effects were negligible for all devices considered here.

S2. DEVICE FABRICATION

All devices were fabricated in an ISO Class 5/6 cleanroom (The Savchenko Centre for Nanoscience, University of Exeter). The chemical vapour deposition (CVD) grown monolayer graphene transferred onto the Si/SiO_2 and quartz wafers was sourced commercially (both from Graphene Laboratories Inc. and Graphene Square Inc.). The monolayer nature of the graphene was confirmed by its distinctive Raman spectrum[3] using a Horiba XploRA with a 532 nm laser. All e-beam lithography was carried out on a Nanobeam NB4 using a beam current of 45 nA and an electron dose of 10 Cm^{-2}. A $6 \times 6\,\mathrm{mm}^2$ etch mask was patterned into a 200 nm layer of PMMA 950K A4 on the surface of CVD graphene, which was then etched using a JLS 80 reactive ion etcher. The plasma power was 15 W and the process gases were Oxygen (5 sccm) and Argon (5 sccm) at a partial pressure of 15 mTorr.

For the three geometries shown at the top of Fig. S1(a), electrodes were defined by electron beam lithography. For the Van der Pauw geometry shown at the bottom, a shadow mask technique was used. Metal contacts were created in a two stage process. First,50 nm of gold (99.99% purity) was deposited on the defined contact areas by thermal evaporation (HHV 306 thermal evaporator). Second, connecting contacts on the SiO_2 surface were patterned and formed of chromium (5 nm) and gold (50 nm). (We found no difference in the transport or acoustic properties between graphene on quartz devices created by electron beam and shadow mask techniques.) Finally, the devices were fixed into multi-pin ceramic leadless chip carriers (Spectrum Semiconductor Materials, Inc.) using conductive silver paint. By ensuring the conductive paint ran up the side of the silicon/silicon dioxide substrates, electrical contact was made to the p-doped silicon layer and enabled gating of the device. The contacts on the devices were connected to the pins of the carrier using 25 μm gold wire (K&S 4123 wedge bonder).

To electrically characterise the back-gated devices, R was measured at μW source powers up to $V_{\mathrm{g}} = +200$ V in liquid helium to determine the gate voltage value, V_{D}, at the Dirac point. Typically, at these powers $V_{\mathrm{D}} \sim +140$ V, which equates to a hole density of $\sim 10^{13}\,\mathrm{cm}^{-2}$. (The mobility was found to be $\sim 660\,\mathrm{cm}^2\mathrm{V}^{-1}\mathrm{s}^{-1}$ around $V_{\mathrm{g}} = 0$.) Neither current annealing[4] in helium gas at room temperature nor annealing in H_2(10%)/Ar at 200 °C shifted this value significantly indicating that the source of doping was likely to be impurities trapped between the graphene and SiO_2.[5]

S3. CONTACT RESISTANCE

The electrode geometry for the back-gated and top gated FETs is shown in Fig. S1(a). Apart from the variation in the resistance between devices with these geometries ($R \sim 500-20,000\,\Omega$ for devices with peripheral electrodes and $R \sim 10-500\,\Omega$ for interdigitated electrodes) we observed no effect of geometry on the sound production (see figure 2a). A low contact resistance was a key attribute of the devices. (A significant contact resistance would cause localised high power dissipation leading to failure of the contact.) This ensured that the thermoa-

coustic generation was confined to the graphene and that our absolute value of R and its temperature dependence of were accurate. The contact resistance was determined through comparative measurements of the two- and four-terminal resistance of multi-terminal devices. If we assume that the conductance of the interface between the graphene and the gold scales with the interfacial area, from these devices we can calculate the conductance per unit area of interface. This was found experimentally to be $\sim 0.45\,\mathrm{S\,mm^{-2}}$. For a two-terminal interdigitated device, a direct calculation was not possible. However, the fabrication method being identical to that of the multi-terminal devices allowed us estimate it. The interfacial area of these devices was $8.2\,\mathrm{mm^2}$ leading to a total contact resistance of $0.3\,\Omega$.

S4. THERMOACOUSTIC MEASUREMENT CIRCUIT

A simplified schematic of the measurement circuit is shown in Fig. S1(b). Voltages at points A, B and (C−D) were measured by lock-in amplifiers locked to the source signal. To obtain the sound pressure spectra, the frequency of the source was stepped in 20 Hz increments over the spectral range. Typically, each full spectrum was measured over 1-4 h to avoid any transients in the signals. Both spectra with increasing and decreasing frequency were measured and compared: within the 20 Hz resolution of the experiments, these were found to be identical. All voltages and sound pressures were measured as rms values.

In order to determine if the power remained constant over the spectral range of the experiment, we modelled and analysed the frequency and phase responses of the circuit. We assumed the high power resistor, $R_\mathrm{s} = 1\,\mathrm{k\Omega}$, in series with the device was purely resistive; the device was assumed to be equivalent to a resistor and capacitor in parallel of resistance R and capacitance C, respectively. The lock-in amplifiers remove the time dependence of the signal so we have:

$$v_\mathrm{A} = V_\mathrm{A}\mathrm{e}^{j\psi_\mathrm{A}} \;,\; v_\mathrm{B} = V_\mathrm{B}\mathrm{e}^{j\psi_\mathrm{B}} \;,$$

where the complex voltages $v_\mathrm{A,B}$ have magnitudes $V_\mathrm{A,B}$ and phase shifts $\psi_\mathrm{A,B}$. The frequency response

$$\left|\frac{v_\mathrm{B}}{v_\mathrm{A}}\right| = \frac{V_\mathrm{B}}{V_\mathrm{A}} = \frac{1}{\sqrt{(R_\mathrm{s}/R+1)^2 + (\omega C R_\mathrm{s})^2}} \;,$$

and the phase response

$$(\psi_\mathrm{B} - \psi_\mathrm{A}) = \Delta\psi = \tan^{-1}\left(-\frac{\omega C R_\mathrm{s}}{R_\mathrm{s}/R+1}\right) \;.$$

The power dissipated,

$$P = \frac{V_\mathrm{B}^2}{R} = \frac{V_\mathrm{B}^2}{R_\mathrm{s}}\left(\sqrt{(V_\mathrm{A}/V_\mathrm{B})^2 - (\omega C R_\mathrm{s})^2} - 1\right) \;.$$

Parameter	Value	Ref.
ρ_a	$1.2\,\mathrm{kg\,m^{-3}}$	7
ρ_s	$2400\,\mathrm{kg\,m^{-3}}$	7
ρ_g	$2236\,\mathrm{kg\,m^{-3}}$	8*
c_a	$1005\,\mathrm{J\,kg^{-1}\,K^{-1}}$	7
c_s	$720\,\mathrm{J\,kg^{-1}\,K^{-1}}$	7
c_g	$700\,\mathrm{J\,kg^{-1}\,K^{-1}}$	9
κ_a	$0.026\,\mathrm{W\,K^{-1}\,m^{-1}}$	7
κ_g	$600\,\mathrm{W\,K^{-1}\,m^{-1}}$	10†
η	$19.8\,\mu\mathrm{N\,s\,m^{-2}}$	2
h_c	$20\,\mathrm{W\,m^{-2}\,K^{-1}}$	1
Pr	0.7	7

TABLE S1. Parameters used in the calculation of the thermoacoustic response. *assumed from specific surface area. †value on substrate.

The model and measurements of $V_\mathrm{A,B}$ and $\Delta\psi$ were found to agree well. For the back-gated FETs, we found the capacitance, $C \approx 90\,\mathrm{pF}$. This capacitance is a very small fraction of the total signal ($\Delta\psi$ changed by 5° over the full spectral range): it originates in roughly equal measure from the experimental coaxial wiring (determined from measurements where the device was replaced by a $1\,\mathrm{k\Omega}$ metal film resistor and by an open circuit) and the gate–channel capacitance of the device. The resistance of the device and the power can then be calculated from the model: both R and P vary by $< 0.5\%$ over the full spectral range. For the top-gated FETs, the gate capacitance per unit area was $\sim 20\,\mathrm{nF\,mm^{-2}}$,[6]. Again, we can use this to correct for signal loss. With this correction (which only affects the data above $\sim 20\,\mathrm{kHz}$) the data match the expected dependence well.

S5. PARAMETERS USED IN THE FITS

The parameters used in the fits shown in figure 2 of the main text are given in Table S1. There are two less well defined parameters to consider: κ_s for the back-gated FETs where the substrate has two distinct layers, and the effect of the electrolyte forming the top gate. First, as $e_\mathrm{s} \gg \max\{e_\mathrm{a}, e_\mathrm{g}^*\}$ for the back-gated devices, most of the Joule heat generated in the graphene is transferred to the substrate. As a result, the thermal properties of the substrate play a significant role. The value of κ_s used for the SiO_2/p^+Si substrate in the model takes this layering into account. The effective thermal conductivity of the substrate is calculated from the thermal conductance K of its two layers in series:

$$\frac{S_\mathrm{g}}{K_\mathrm{s}} = \frac{d_\mathrm{s}}{\kappa_\mathrm{s}} = \frac{d_\mathrm{Si}}{\kappa_\mathrm{Si}} + \frac{d_\mathrm{SiO2}}{\kappa_\mathrm{SiO2}} \;,$$

where $d_\mathrm{s} = d_\mathrm{Si} + d_\mathrm{SiO2}$ is the total skin depth, $\kappa_\mathrm{Si} = 120\,\mathrm{W\,K^{-1}\,m^{-1}}$ for doped silicon[11] and $\kappa_\mathrm{SiO2} =$

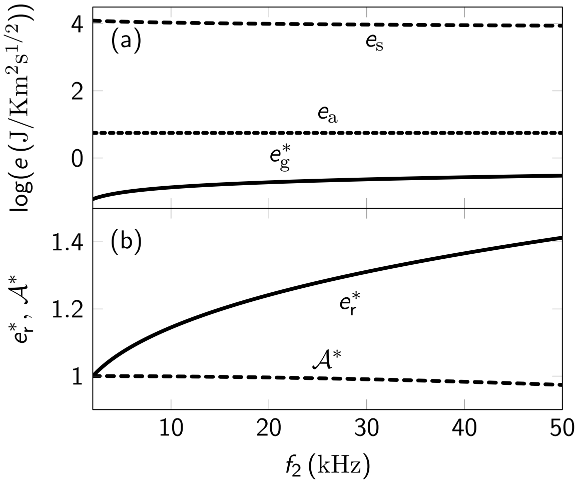

FIG. S2. (a) Frequency dependences of the effusivities of the air (dotted line) and SiO_2/p^+Si substrate (dashed line), and the effective effusivity of graphene (solid line). (b) The parameters e_r (solid line) and $\mathcal{A}$ (dashed line) of Eqn. S2 normalised (*) to their values at $f_2 = 2\,\mathrm{kHz}$ and $r = 1\,\mathrm{m}$: $e_r(2\,\mathrm{kHz}) = 0.00045$, $\mathcal{A}(2\,\mathrm{kHz},\,1\,\mathrm{m}) = 0.99996$.

$1.4\,\mathrm{W\,K^{-1}\,m^{-1}}$ for silicon dioxide. The skin depth of silicon dioxide is much greater than its physical thickness for the range of frequencies considered, therefore,

$$\kappa_s(\omega_2) = \frac{d_{\mathrm{Si}}(\omega_2) + d_{\mathrm{SiO2}}}{d_{\mathrm{Si}}(\omega_2)/\kappa_{\mathrm{Si}} + d_{\mathrm{SiO2}}/\kappa_{\mathrm{SiO2}}}\ .$$

Second, if we assume a simple extension of the model where the electrolyte layer acts as another thermal absorber then it will enter into the model through an additional effusivity term in the denominator of e_r. If we take values for the thermal conductivity, heat capacity and density for a typical polymer[12], then we should expect an effusivity of the order $10^3\,\mathrm{J\,K^{-1}\,m^{-2}\,s^{-0.5}}$.

The speed of sound in air is given by

$$v_a(T_{eq}) = \sqrt{\gamma R T_{eq}/M} \approx 20\sqrt{T_{eq}}\,\mathrm{m\,s^{-1}}\ ,$$

where R is the universal gas constant, M is the molecular weight of the gas and $\gamma = c_p/c_v$ is the adiabatic constant. Experimentally, we found that $T_{eq} = aP + T_0$ where $a \approx 6\,\mathrm{K/W}$. From this we can determine v_a given the power P within the distance $\sim r_0$ from the device surface. The air temperature will gradually decay from T_{eq} close to the device to T_0: in our fits, whether we use $v_a(T_{eq})$ or $v_a(T_0)$ is not significant for powers below 20 W.

Figure S2(a) shows how the effusivities of air and the substrate and the effective effusivity of graphene vary as a function of the frequency. It can be seen that the effusivity of the substrate dominates by several orders of magnitude. The comparatively small values for graphene are due entirely to its thickness. The effusivities combine to form the parameter e_r in Eqn. S2 and this is shown together with $\mathcal{A}$ in Fig. S2(b). The attenuation associated with $\mathcal{A}$ varies minimally over the full spectral range

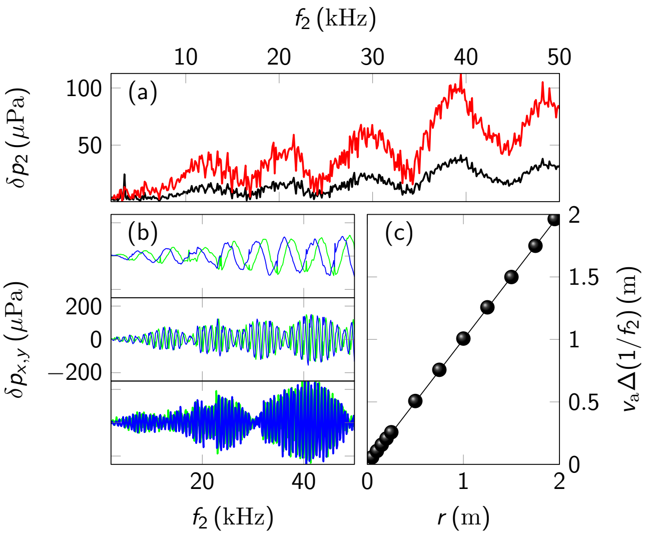

FIG. S3. (a) Sound pressure as a function of frequency for a back-gated FET (measured at $r = 0.25\,\mathrm{m}$) at source powers of 0.28 W (black) and 0.78 W (red). (b) The in-phase (δp_x; green) and out-of-phase (δp_y; blue) components of the sound pressure generated by device Ac8 as a function of frequency for different device–microphone separations: $r = 50\,\mathrm{mm}$ (top), 250 mm (middle), and 500 mm (bottom). All vertical axes are the same range. (c) Separation calculated (symbols) from the Fourier transforms of the sound pressure components measured at various values of r using equation (S3) as a function of the actual separation. The line is the function $r = r$.

whereas e_r varies by $\sim 40\%$. This variation originates from the frequency dependence of κ_s. (It is not significant compared to the overall frequency dependence of δp and a good fit to the experimental sound spectrum can be obtained by taking a frequency-independent value for κ_s of $\sim 70\,\mathrm{W\,K^{-1}\,m^{-1}}$.)

S6. SPECTRAL ANALYSIS

The agreement between the model and experimental sound pressure spectra is generally good, but there are deviations. First, at frequencies up to $\sim$ 10 kHz the model predicts the sound pressure to increase linearly whereas the data can show super-linear behaviour. Second, as r is increased pronounced dips in the pressure can be observed, Fig. S3(a). Third, reproducible fine structure occurs over the full spectral range. These deviations were observed to have no significant dependence on R or P. By measuring the charge transport simultaneously with the sound pressure, we found that both R and P vary monotonically with frequency by less than 0.5% over the full spectral range. Finally, from experiments we found no evidence that the dips were due to focussing[13] or an off-axis effect of $\mathcal{H}$, the directional factor.[14]

The lock-in technique allows investigation of the structure of $\delta p_2(f_2)$ by separation of its components that

are in-phase (x) or out-of-phase (y) with the source signal. By recording both the magnitude and phase of δp_2, the x- and y-components can be determined, Fig. S3(b). The components have an equal, well-defined period in the frequency that varies with r. In addition, there are two envelopes. One increases linearly with frequency, as expected for a thermoacoustic source: $(\delta p_2)^2 = (\delta p_x)^2 + (\delta p_y)^2$. The other is oscillatory and indicative of a form of beating. It can now be seen from where the structure of the magnitude of the signal originates: the dips correspond to minima in the beats and the super-linear behaviour results from the envelope of the first beat. By taking the Fourier spectra of the data, we determined the period of the fine structure. If this structure is related to the sound wavefronts passing the microphone then we would expect

$$\Delta\left(1/f_2\right) = r/v_{\mathrm{a}} \,. \tag{S3}$$

Figure S3(c) shows the agreement found between experiment and Eqn. S3. The reason for the beating is less obvious. It must originate from a path-length difference and, therefore, could be expected from reflections. However, as the beat period was found to depend on the absolute value of r this could not be the case. In addition, it had a very weak yet discernible power dependence, as can be seen in figure S3(a). At separations, $r < 0.1$ m, the beat period was comparable to the period of the fine structure so was not resolved. Nevertheless, it did appear to be a simple artefact: by tangentially shifting the microphone away from the centre of the device by an amount several times the width of the device, the beating was significantly reduced.

S7. DEVICE TEMPERATURE

The equilibrium temperature, T_{eq}, of the system will occur when the power generated in the device is balanced by the power lost as heat to the air through convection (con) and radiation (rad):

$$P_{\mathrm{con}} = h_c S_{\mathrm{D}}(T_{\mathrm{eq}} - T_0)\ ,$$
$$P_{\mathrm{rad}} = \sigma\epsilon_{\mathrm{D}} S_{\mathrm{D}}(T_{\mathrm{eq}}^4 - T_0^4)\ ,$$

where h_c is the convection constant, σ is the Stefan–Boltzmann constant, S_{D} is the total area of the device, ϵ_{D} is the (average) emissivity of the device and T_0 is the temperature of the air. Therefore, the equilibrium temperature is found from the solution of

$$P = S_{\mathrm{D}}\left(h_c(T_{\mathrm{eq}} - T_0) + \sigma\epsilon_{\mathrm{D}}(T_{\mathrm{eq}}^4 - T_0^4)\right)\ . \tag{S4}$$

This assumes that the power lost as sound is negligible, which is the case as $\mathcal{E} \ll 1$.

Figure S4(a) shows a plot of Eqn. S4 and the two components of the power loss. It can be seen that both the radiative and convective losses are significant in the system.

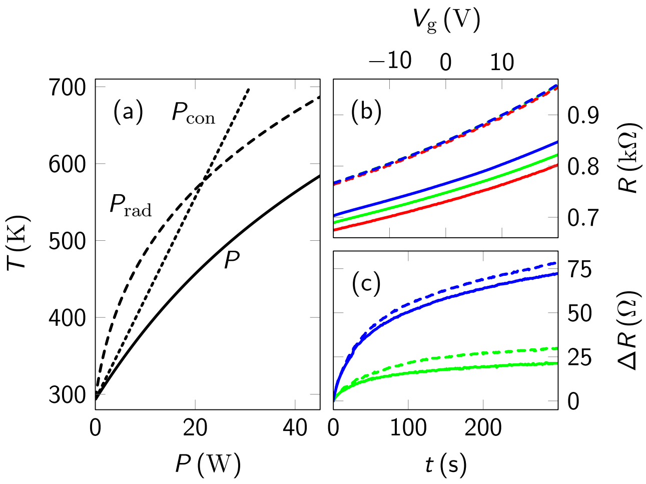

FIG. S4. (a) The variation of temperature with power (solid line) expected from Eqn. S4. The power losses via radiation (dashed line) and convection (dotted line) are also shown. (b) Resistance (R) of a back-gated FET as a function of gate voltage (V_{g}) under ambient (solid lines) and liquid helium immersion (dashed lines) conditions and different source powers: 0.03 W (red), 0.27 W (green), 0.75 W (blue). (c) Transient resistance change upon immediately applying 0.27 W (green) and 0.75 W (blue) source power under ambient (solid lines) and vacuum (dashed lines) conditions at $V_{\mathrm{g}} = 0$.

Figure S4(b) shows measurements of $R(V_{\mathrm{g}})$ of a back-gated FET, both in liquid helium and ambient conditions, at different source powers. When immersed in liquid helium, R varied negligibly as a function of P over the full gate voltage range. In contrast, in ambient conditions a significant change was observed. This suggests that in liquid helium the substrate temperature is pinned and all Joule heat is absorbed by the liquid as latent heat. As a result, there is no heat-dependent change of the resistance of the graphene. In ambient conditions, the Joule heat can be absorbed by the substrate (as air is such a poor thermal conductor) and raise its temperature.[15] In this case, we observe a temperature-dependence of R due to the thermal anchoring of the graphene to the substrate surface.[16]

Figure S4(c) shows how the resistance of a back-gated FET changed as a function of time upon a step change in the source power from zero. By comparison with Fig. S4(b), it is clear that the device temperature increased, but it did so at a relatively slow rate (dictated by the volume of material heated in the device). The experiment was repeated under low vacuum conditions ($\sim 10^{-4}$ mbar). It can be seen that in this case, the resistance change was greater indicating that the device temperature was higher than it was in ambient conditions. This provided direct evidence that convective heat loss occurs in the air layer above the device. Figure S4(a) highlights how the removal of this loss mechanism in the transient measurements causes an additional increase in the device temperature for a fixed power.

The significant heating in devices at source powers > 10 W (figure 2c) caused no degradation of the transistor performance: $R(V_g)$ curves taken before and after such a thermal cycle were found to be identical, despite the high temperatures attained (and, in some cases, observed melting/solidifying of the silver paint used to electrically contact the gate).

The power lost as sound in the air,

$$\mathcal{P} = \int_S \frac{\delta p^2}{z} \mathrm{d}S \approx \frac{9\mathcal{E}^2}{8\pi\rho_\mathrm{a} v_\mathrm{a}^5} (fP)^2 \,, \tag{S5}$$

where $z = \rho_\mathrm{a} v_\mathrm{a}$ is the specific acoustic impedance and S is the area of the wavefront at radius r. Even though the efficiency, $\mathcal{P}/P \sim 1\,\mathrm{pW/W}$ appears to be very low, the sound produced is at an audible level (above 0 dB) above $1-10$ kHz per Watt of source power. Equation S5 shows that the efficiency increases quadratically with frequency, power and relative effusivity. As source power can be controlled directly through the applied gate voltage in a graphene FET (figure 5), so too can the efficiency.

S8. NONLINEARITY AND HETERODYNING

Consider a current bias consisting of a dc and ac (sinusoidal) component: $I = I_\mathrm{dc} + I_\mathrm{ac}\cos\omega t$. For clarity, we define $\xi_n \equiv \cos(n\omega t)$. If the charge transport is considered nonlinear to second order in the current, then the resulting voltage

$$\begin{aligned} V(t) &= V_\mathrm{dc} + \left.\frac{\mathrm{d}V}{\mathrm{d}I}\right|_{I_\mathrm{dc}} I_\mathrm{ac}\xi_1 + \frac{1}{2}\left.\frac{\mathrm{d}^2V}{\mathrm{d}I^2}\right|_{I_\mathrm{dc}} I_\mathrm{ac}^2\xi_1^2 \\ &= R_0 I_\mathrm{dc} + R_1 I_\mathrm{ac}\xi_1 + \tfrac{1}{2}R_2 I_\mathrm{ac}^2\xi_1^2 \,, \end{aligned}$$

where R_0 is the dc resistance, R_1 is the differential resistance, and R_2 is the second-differential resistance. The power

$$P = IV = (I_\mathrm{dc} + I_\mathrm{ac}\xi_1)\left(R_0 I_\mathrm{dc} + R_1 I_\mathrm{ac}\xi_1 + \tfrac{1}{2}R_2 I_\mathrm{ac}^2\xi_1^2\right) \,.$$

If we expand this out, we find

$$P = P_0 + P_1\xi_1 + P_2\xi_2 + P_3\xi_3 \,,$$

where the components

$$\begin{aligned} P_0 &= R_0 I_\mathrm{dc}^2 + \left(R_1 + \frac{1}{2}R_2 I_\mathrm{dc}\right)\frac{I_\mathrm{ac}^2}{2} \,, \\ P_1 &= (R_0 + R_1) I_\mathrm{dc} I_\mathrm{ac} + 3R_2\left(\frac{I_\mathrm{ac}}{2}\right)^3 \,, \\ P_2 &= \left(R_1 + \frac{1}{2}R_2 I_\mathrm{dc}\right)\frac{I_\mathrm{ac}^2}{2} \,, \\ P_3 &= R_2\left(\frac{I_\mathrm{ac}}{2}\right)^3 \,. \end{aligned}$$

Now consider the case of a bias current consisting of two ac components, $I = I_\mathrm{A}\cos\omega_\mathrm{A}t + I_\mathrm{B}\cos\omega_\mathrm{B}t$. Again, for clarity we define $\xi_\mathrm{X} \equiv \cos(\omega_\mathrm{X}t)$. The resulting power,

$$\begin{aligned} P =& R_1\left((I_\mathrm{A}\xi_\mathrm{A})^2 + (I_\mathrm{B}\xi_\mathrm{B})^2 + 2I_\mathrm{A}I_\mathrm{B}\xi_\mathrm{A}\xi_\mathrm{B}\right) \\ &+ \tfrac{1}{2}R_2\left((I_\mathrm{A}\xi_\mathrm{A})^3 + (I_\mathrm{B}\xi_\mathrm{B})^3\right) \\ &+ \tfrac{3}{2}R_2\left(I_\mathrm{A}^2 I_\mathrm{B}\xi_\mathrm{A}^2\xi_\mathrm{B} + I_\mathrm{A}I_\mathrm{B}^2\xi_\mathrm{A}\xi_\mathrm{B}^2\right) \,. \end{aligned}$$

Upon expanding this, it will be found that the only term that has components at the heterodynes $\omega_\mathrm{A} \pm \omega_\mathrm{B}$ is the single term containing $\xi_\mathrm{A}\xi_\mathrm{B}$. Therefore, irrespective of whether the conductor is linear or not, the power at the heterodynes is

$$P_{\mathrm{A}\pm\mathrm{B}} = R_1 I_\mathrm{A} I_\mathrm{B} \,.$$

[1] M. Daschewski, R. Boehm, J. Prager, M. Kreutzbruck, and A. Harrer, Journal of Applied Physics **114**, 114903 (2013).

[2] L. E. Kinsler, A. R. Frey, A. B. Coppens, and J. V. Sanders, *Fundamentals of Acoustics* (Wiley, 2000), ISBN 978-0-047-84789-2.

[3] A. C. Ferrari and D. M. Basko, Nature Nanotechnology **8**, 235246 (2013).

[4] J. Moser, A. Barreiro, and A. Bachtold, Applied Physics Letters **91**, 163513 (2007).

[5] C. W. Jang, J. H. Kim, J. M. Kim, D. H. Shin, S. Kim, and S.-H. Choi, Nanotechnology **24**, 405301 (2013).

[6] A. Das, S. Pisana, B. Chakraborty, S. Piscanec, S. K. Saha, U. V. Waghmare, K. S. Novoselov, H. R. Krishnamurthy, A. K. Geim, A. C. Ferrari, et al., Nature Nanotechnology **3**, 210 (2008).

[7] J. H. Lienhard IV and J. H. Leinhard V, *A Heat Transfer Textbook* (Phlogiston Press, 2015), 4th ed., URL `http://ahtt.mit.edu`.

[8] Y. Zhu, S. Murali, W. Cai, X. Li, J. W. Suk, J. R. Potts, and R. S. Ruoff, Advanced Materials **22**, 3906 (2010).

[9] E. Pop, V. Varshney, and A. K. Roy, MRS Bulletin **37**, 1273 (2012).

[10] J. H. Seol, I. Jo, A. L. Moore, L. Lindsay, Z. H. Aitken, M. T. Pettes, X. Li, Z. Yao, R. Huang, D. Broido, et al., Science **328**, 213 (2010).

[11] M. Asheghi, K. Kurabayashi, R. Kasnavi, and K. E. Goodson, Journal of Applied Physics **91**, 5079 (2002).

[12] X. Xie, D. Li, T.-H. Tsai, J. Liu, P. V. Braun, and D. G. Cahill, Macromolecules **49**, 972 (2016).

[13] J. W. Suk, K. Kirk, Y. Hao, N. A. Hall, and R. S. Ruoff, Advanced Materials **24**, 6342 (2012).

[14] V. Vesterinen, A. O. Niskanen, J. Hassel, and P. Helistö, Nano Letters **10**, 5020 (2010).

[15] M. Freitag, M. Steiner, Y. Martin, V. Perebeinos, Z. Chen, J. C. Tsang, and P. Avouris, Nano Letters **9**, 1883 (2009).

[16] P. J. Hale, S. M. Hornett, J. Moger, D. W. Horsell, and E. Hendry, Phys. Rev. B **83**, 121404 (2011).
